# Could the implementation of a trauma checklist improve quality of care?

**DOI:** 10.1186/1757-7241-23-S2-A13

**Published:** 2015-09-11

**Authors:** Elizabeth Ashton

**Affiliations:** 1 Peninsula College of Medicine and Dentistry, Plymouth, UK

## Background

Medical error in trauma care remains common [1]. Checklists are a cognitive aid that can be employed to standardise practice and minimise error. Their usage is ubiquitous in other high-intensity professions, such as aviation. Following the success of the World Health Organisation's surgical safety checklist they are now in the process of developing a trauma care checklist. My aim was to evaluate whether a checklist could be applied to the trauma setting to facilitate high quality, standardised care.

## Method

1. Audit the prevalence of human factors during trauma calls in a major trauma centre in the UK

2. Literature review of the use of checklists in medicine

## Results

• At least one incidence of negative human factors affecting trauma team performance was observed during each trauma call with the average incidence being three times per resuscitation.

• Evidence suggests that poor communication is the leading cause of medical error. This was corroborated by my audit findings.

• A review of literature shows that checklists in are effective tools for standardising care by reducing error and improving compliance with guidelines [2].

## Conclusion

It is essential that organisational safety culture is addressed and the subject of human error is acknowledged to improve care provision and enhance patient safety. Checklists represent a promising method of tackling this issue that should be used synergistically with existing management strategies.
